# The ability of captive spider monkeys, *Ateles geoffroyi,* to visually discriminate between different sizes of food and of non-edible objects

**DOI:** 10.1038/s41598-025-06479-8

**Published:** 2025-06-20

**Authors:** Anne-Sophie van Herwijnen, Laura Teresa Hernández Salazar, Matthias Laska

**Affiliations:** 1 IFM Biology, Linköping University, SE-581 83 Linköping, Sweden; 2 Instituto de Neuroetologia, Universidad Veracruzana, C.P. 91000 Xalapa, Mexico

**Keywords:** Visual size discrimination, Geoffroy’s spider monkey, *Ateles geoffroyi*, Animal behaviour, Animal physiology, Zoology

## Abstract

Field studies suggest that the size of potential food items plays an important role in the food selection behavior of nonhuman primates. However, there is only limited knowledge about how good primates are at visually discriminating between the size of three-dimensional objects of the same kind and shape. We therefore conducted two experiments on ten adult spider monkeys: a two-choice test based on spontaneous preferences with differently-sized pieces of food of the same kind and shape, and a two-choice test based on an operant conditioning procedure in which the animals were trained to choose the larger one of two cube-shaped wooden blocks. We found that the spider monkeys displayed a robust and spontaneous preference for the larger one of two simultaneously presented pieces of food of the same kind when the size difference was 11% or larger. This was true with cube-shaped food pieces as well as with ball-shaped and hemisphere-shaped food pieces. Considering that the normal size variation among fully ripe fruits of a given plant species is at least 10% this suggests that spider monkeys are as picky as necessary to make size-based food choices in line with optimal foraging theory. We also found that the spider monkeys successfully discriminated between two simultaneously presented wooden cubes when their edge length differed by only 2 mm which was the smallest size difference tested. Thus, they displayed a well-developed ability to visually discriminate between three-dimensional non-edible objects of the same kind and shape which is at least as good as that of other nonhuman primate species tested previously on similar tasks. Our findings support the notion that frugivory may have favored the evolution of cognitive abilities in the physical domain which may, possibly, include the ability to perform fine visual size discriminations.

**Supplementary Information:**

The online version contains supplementary material available at 10.1038/s41598-025-06479-8.

## Introduction

Animals need to efficiently find and choose between available food sources in order to meet their requirements of critical nutrients and metabolic energy^1^. To this end, animals utilize the chemical and physical properties of potential food items such as their taste and smell as well as their color, size, shape, hardness, and texture, to make informed decisions about their palatability and nutritional value^2^.

Numerous studies have demonstrated that frugivorous primates use all their senses for evaluating potential food items^3,4^. The odor of a fruit, for example, has been shown to be an honest signal informing the animal about its degree of ripeness and thus about its nutritional value^5,6^. The taste of a fruit usually allows an animal to draw conclusions about its caloric value and/or its toxicity, although some plants evolved non-caloric sweeteners to attract primates as seed dispersers without providing metabolic energy in the form of carbohydrates^7,8^.

Fruits often change color across the process of maturation and thus provide a visual cue to learn about their state of ripeness^9^. However, there are also fruits consumed by primates which do not change color during ripening^10^. Instead, fruits such as figs (genus *Ficus*), for example, do change in both hardness and size and thus allow an animal to base its decision about whether to consume or discard the fruit on its somatosensory and non-color-related visual properties^11^. Among the latter, size is probably the most reliable physical property as fruits inevitably increase in size from the immature to the mature stage^12^. The importance of size for food selection should be obvious considering that the size or volume of a potential food item is generally proportional to its mass and thus to its total amount of nutrients and metabolic energy. Accordingly, fruit size has repeatedly been reported to be a factor in primate food selection^13–16^. However, in this context, it is important to note that fruit selection in primates is not a simple matter of “the bigger, the better”. Rather, at least for certain fruits there seems to be an optimal size for a given primate species for which handling time and mouth gape width are restricting factors^17,18^.

The ability to discriminate between three-dimensional objects based on size differences may also be important for primates outside the context of food selection, for example in social conflict (e.g. when sizing up an intruder), in object manipulation (e.g. when choosing the optimal size of a tool), in sexual selection (e.g. when assessing the degree of sexual swelling in a female conspecific, or when using body size as physical trait for selecting among potential mating partners) and in predator avoidance (e.g. when deciding whether to fight or flee depending on the predator’s body size).

Geoffroy’s spider monkeys (*Ateles geoffroyi*) are highly frugivorous platyrrhines and specialize on ripe fruit^19,20^, including cryptic fruit species which do not change color during ripening^21,22^. Therefore, it should be important for spider monkeys to have a well-developed ability to visually discriminate between fruits of a given species that differ in size in order to maximize their net gain of metabolic energy and critical nutrients^1^. Spider monkeys have also been reported to be territorial and thus should benefit from being able to size up an intruder to decide whether to seek or to avoid physical conflict with a conspecific^23^. Similarly, spider monkeys have been reported to use tools and thus should benefit from choosing the optimal size of a branch, for example, which is then used as a tool^24^. Finally, although female spider monkeys do not show the conspicuous sexual swelling typical for some cercopithecoid and hominoid primates, they have been reported to show a swelling of their unusually large clitoris during midcycle and thus males may increase their inclusive fitness by being able to correctly identify such a sexual swelling^25^.

Frugivory has repeatedly been proposed to foster the evolution of cognitive abilities in the physical domain^26–28^. The complex spatio-temporal distribution of fruit, for example, requires cognitive skills such as mental maps, long-term spatial and sensory memory, and decision-making skills in order to remember the location of patchily distributed food sources, to efficiently predict times and places to forage, and to optimize food selection^29^. This may, possibly, also include an advanced ability to discriminate between different sizes of food.

Considering that the size of three-dimensional objects should be important for spider monkeys in food selection and other behavioral contexts, it was the aim of the present study.

1. to assess the ability of spider monkeys to visually discriminate between different sizes of a given type of food and.
2. to assess their ability to visually discriminate between different sizes of a non-edible three-dimensional object of the same shape.

To this end, we conducted two experiments: a two-choice test based on spontaneous preferences with differently-sized pieces of food of the same kind, and a two-choice test based on an operant conditioning procedure in which the spider monkeys were trained to choose the larger one of two cube-shaped wooden blocks.

Whereas operant conditioning procedures usually allow us to assess the limits of a sensory capability such as size discrimination, spontaneous preference tests only allow for a first and conservative approximation of an animal’s sensory capability because small size differences between food items, for example, may not be behaviorally relevant enough to yield a clear preference, despite the animal’s sensory ability to perceive the size difference in question^30^. However, a spontaneous preference test is more similar to the situation that an animal encounters in the wild during food selection compared to an operant conditioning test which requires an animal to learn an abstract concept.

We predicted that

1. the spider monkeys would display a spontaneous preference for the larger one of two simultaneously presented pieces of food,
2. they would learn to choose the larger one of two simultaneously presented non-edible three-dimensional objects,
3. the spider monkeys would discriminate between smaller size differences in the operant conditioning procedure compared to the smallest size difference for which they display a spontaneous preference.

## Methods

### Animals

The study was carried out with 10 adult Geoffroy’s spider monkeys (*Ateles geoffroyi*). The group consisted of five males and five females, aged between 5 and 20 years, and all born in captivity. The animals were maintained at the field station UMA Doña Hilda Ávila de O’Farrill of the Universidad Veracruzana, located near Catemaco, state of Veracruz, Mexico. They were housed in a roofed enclosure of 20 × 10 × 8 m which was subdivided into ten compartments of equal size. The enclosures were connected by sliding doors which were usually kept open allowing the animals to interact with each other but could be closed for temporary separation of individuals. The animals were exposed to natural light and ambient temperature and provided with fresh fruits and vegetables once per day and occasionally with seeds and edible foliage to complement their diet. The enclosures were equipped with branches, ropes, tires, perches, sleeping boxes and further enrichment designed for climbing, swinging, and resting. The experiments described below were carried out in the morning before feeding and no food deprivation schedule was adopted. The animals had participated in previous studies on their cognitive abilities^28,31^ as well as in sensory discrimination tasks^32^ and were thus accustomed to participating in behavioral tests and to temporary separation. All animals were tested individually to prevent interference from, and distraction by, the other animals.

### Experiment 1: spontaneous preference test - visual size discrimination of food items

We used a two-choice test based on spontaneous preferences to assess the ability of spider monkeys to visually discriminate between different sizes of a given type of food. The animals were simultaneously presented with two pieces of food of the same kind which only differed in size. In each trial, the animal was allowed to choose only one of the two pieces of food, thus it had to make a decision based on its spontaneous preference. By systematically varying the size of the food pieces we determined the minimum size difference for which the spider monkeys still displayed a spontaneous preference for the larger one of two pieces of food.

### Experimental design

At the start of each session, an animal was called to come to the front of the enclosure where it sat on a platform at the mesh. The animal was then presented with two pieces of honeydew melon (*Cucumis melo*) placed on a tray (36 × 26 × 1.5 cm) at a distance of 20 cm from each other. To ensure that the animal paid attention, the tray was first presented just out of the animal’s reach for a few seconds. Then the tray was moved forward so that the animal could reach through the mesh and take one of the two melon pieces. The tray was then moved downwards and out of reach to prevent the animal from taking the other melon piece. The animal’s decision for the larger or the smaller one of the two melon pieces was recorded.

Ten such presentations constituted a session, and one or two sessions were performed per day and animal. Care was taken to present the larger one of the two simultaneously presented melon pieces equally often on the right and on the left side, respectively, and it was only presented on the same side for a maximum of three times in a row to control for potential side biases. Further, care was taken to present the tray in such a way that the two melon pieces were to the left and to the right of the animal’s midline, respectively, to control for possible hand preferences.

Six sessions each of the following five conditions were performed with each animal in the order mentioned below:

(A) Cube-shaped melon pieces of three different sizes (small: 10 x 5 x 5 mm; medium: 15 x 15 x 10 mm; and large: 15 x 15 x 20 mm) were presented to the animals. A commercial fruit cutter (Ikea, Sweden) was used to cut melon pieces to these dimensions. Each of the three possible stimulus combinations (small *versus* medium, small *versus* large, and medium *versus* large) was presented in a pseudo-randomized order for a total of 20 times.
(B) Cube-shaped melon pieces of varying intermediate or smaller sizes were cut by hand from the three sizes mentioned above and presented to the animals.
(C) Ball-shaped melon pieces of two different sizes (small: 15 mm diameter; large: 30 mm diameter) were presented to the animals. Spoon-shaped ice cream scoops (Ikea, Sweden) were used to cut the ball-shaped melon pieces to these dimensions.
(D) Ball-shaped melon pieces of varying intermediate or smaller sizes were cut by hand from the two sizes mentioned above and presented to the animals.
(E) Hemisphere-shaped melon pieces of varying sizes were cut by halving ball-shaped melon pieces of the sizes mentioned above and presented to the animals with the flat surface down.

As it was difficult to cut the melon pieces to exact dimensions by hand, they were weighed instead, using a scale (Ruhhy, Poland) to a precision of 0.01 g. (Please note that the weight of the melon pieces is directly proportional to their volume, that is, to their size.)

The rationale for using the three shapes of food included in this study was as follows: the cube-shaped food pieces allowed us to directly compare the animals’ performance in this experiment with their performance in the second experiment with cube-shaped non-edible objects. The ball-shaped food pieces clearly resemble the shape of the fruits that spider monkeys feed on in the wild whereas the cube-shaped food pieces do not which allowed us to assess whether familiarity with or naturalness of the food shapes may affect the animals’ performance. The hemisphere-shaped food pieces allowed us to gain further insight into whether the presence or absence of clear edges on the food pieces may affect the animals’ performance.

### Experiment 2: **operant conditioning test - visual size discrimination of wooden blocks**

We used a two-choice test based on an operant conditioning procedure to assess the ability of spider monkeys to visually discriminate between different sizes of a non-edible three-dimensional object. The animals were simultaneously presented with two cube-shaped wooden blocks which only differed in size. They were trained that choosing the larger one of the two wooden blocks was food-rewarded whereas choosing the smaller one was not. By systematically varying the size of the wooden blocks we determined the minimum size difference for which the spider monkeys were still able to correctly choose the larger one of the two wooden blocks.

Due to health problems of one spider monkey, only nine of the ten animals used in Experiment 1 also participated in Experiment 2.

### Experimental design

Here, too, at the start of each session, an animal was called to come to the front of the enclosure where it sat on a platform at the mesh. The animal was then presented with a test apparatus which consisted of a metal bar of 50 × 6 cm, with two PVC boxes (5 × 5 × 5 cm) attached to the bar at a distance of 22 cm. The boxes were fitted with hinged metal lids (6 cm x 6.8 cm) onto which square-shaped PVC plates (3 × 3 × 0.3 cm) bearing cube-shaped wooden blocks of nine different sizes could be attached. The wooden blocks had an edge length of 10, 12, 14, 15, 16, 18, 20, 25, and 30 mm, respectively.

To ensure that the animal paid attention, the apparatus was first presented just out of the animal’s reach for a few seconds. Then the apparatus was moved forward so that the animal could reach through the mesh and open one of the boxes by flipping up the lid. In the case of a correct response (i.e. choosing the box bearing the larger one of the two wooden blocks), the animal then retrieved the food reward (a Kellogg’s honey loop) from the box, and in case of an incorrect response (i.e. choosing the box bearing the smaller one of the two wooden blocks), the animal found that the corresponding box was empty. The apparatus was then moved downwards and out of reach to prevent the animal from opening the other box. The animal’s decision for the larger one or the smaller one of the two wooden blocks was recorded as a correct or an incorrect response, respectively.

Ten such presentations constituted a session, and one or two sessions were performed per day and animal. Care was taken to present the larger one of the two simultaneously presented wooden blocks equally often on the right and on the left side, respectively, and it was only presented on the same side for a maximum of three times in a row, to control for potential side biases. Further, care was taken to present the apparatus in such a way that the two boxes bearing the wooden blocks were to the left and to the right of the animal’s midline, respectively, to control for possible hand preferences.

The following five conditions were performed with each animal in the order mentioned below, adopting an iterative process in which the size difference between the wooden blocks became smaller with each successive condition:

(A) Here, the stimulus combination with the largest size difference of the wooden blocks (30 *versus* 10 mm edge length) was presented to the animals. This condition was also the training phase in which the animals had to learn the concept of the task, that is, to choose the larger one of the two simultaneously presented wooden blocks in order to get a food reward.
(B) Here, one novel size of a wooden block (20 mm edge length) was introduced. This resulted in two novel stimulus combinations (30 *versus* 20 mm, and 20 *versus* 10 mm edge length) that were presented to the animals.
(C) Here, two novel sizes of a wooden block (12 mm, and 25 mm edge length) were introduced. Three novel stimulus combinations (12 *versus* 25 mm, 12 *versus* 20 mm, and 25 *versus* 20 mm) were presented to the animals.
(D) Here, one novel size of a wooden block (15 mm edge length) was introduced. Three novel stimulus combinations of wooden blocks differing by 5 mm in edge length were presented to the animals (15 *versus* 10 mm, 20 *versus* 15 mm and 30 mm *versus* 25 mm).
(E) Here, three novel sizes of a wooden block (14 mm, 16 mm, and 18 mm edge length) were introduced. Three novel stimulus combinations of wooden blocks differing by 2 mm in edge length were presented to the animals (16 *versus* 14 mm, 18 *versus* 16 mm, and 20 *versus* 18 mm).

The criterion for the initial learning of the concept that choosing the larger one of the two simultaneously presented wooden blocks was rewarded was set at 80% correct choices (corresponding to 24 out of 30 correct choices) across three consecutive sessions (two-tailed binomial test, *p* < 0.01). Based on the number of sessions that the animals needed on average to learn the first stimulus combination (6 sessions with the wooden blocks of 30 *versus* 10 mm edge length), a maximum of 12 sessions were performed per animal with the more difficult stimulus combinations. The scores of the last three sessions per stimulus combination was taken as the measure of performance.

### Data analysis

The two-tailed binomial test was used to assess whether the animals’ choices for the larger one of two simultaneously presented pieces of food (Experiment 1) or wooden blocks (Experiment 2) differed from chance.

The Spearman rank-correlation test was used to assess whether the proportion of decisions for the larger piece of food (Experiment 1) or wooden block (Experiment 2) significantly correlated positively with size difference of the discriminanda.

The Mann-Whitney U-test for independent samples was used to assess whether females and males differed from each other in their discrimination performance.

The Friedman repeated measures ANOVA, followed by pairwise post-hoc Wilcoxon tests for related samples, was used to assess whether the animals’ choices for the larger one of two simultaneously presented pieces of food differed as a function of shape.

The Kruskal-Wallis test, that is, a non-parametric one-way ANOVA, followed by pairwise Dunn’s tests, was used to assess whether the animals’ choices for the larger one of two simultaneously presented pieces of food differed as a function of edge length difference.

## Results

### Experiment 1: spontaneous preference test - visual size discrimination of food items

**Fig. 1:**
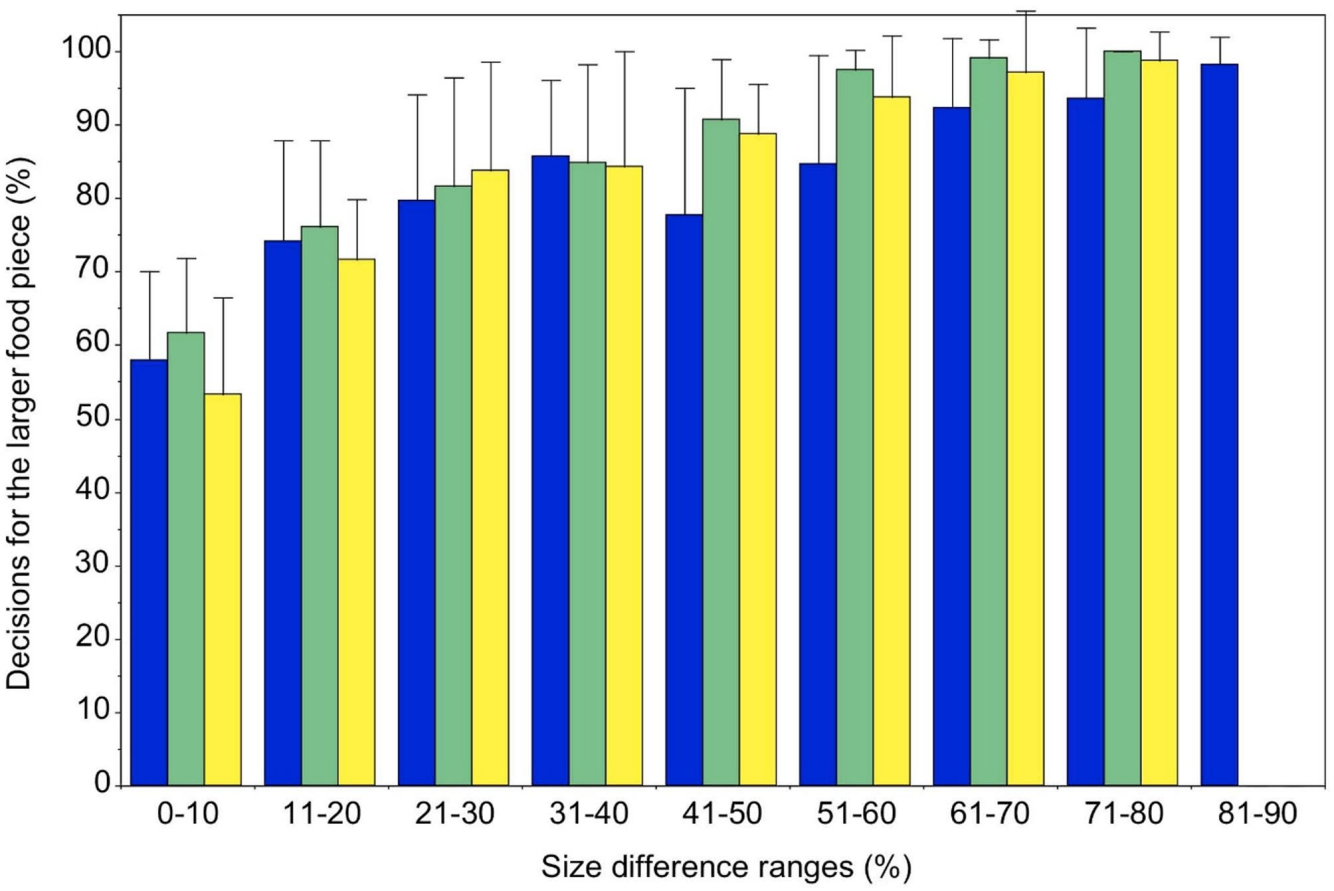
Mean (±SD) percentages of decisions for the larger one of two simultaneously presented food pieces (blue: cube-shaped; green: ball-shaped; yellow: hemisphere-shaped food pieces) for the group of spider monkeys (*n* = 10), subdivided by size difference ranges (%).

The spider monkeys, as a group, displayed a statistically significant preference for the larger one of two simultaneously presented pieces of food of the same kind when the size difference was 11% or more (Fig. 1). This was true with cube-shaped food pieces as well as with ball-shaped and hemisphere-shaped food pieces (Binomial test, *p* < 0.01 with all three shapes). With all three shapes of food pieces, the proportion of decisions for the larger food piece significantly correlated positively with size difference (Spearman test, cubes: r_s_ = 0.92, *p* < 0.001; balls: r_s_ = 0.99, *p* < 0.001; hemispheres: r_s_ = 0.99, *p* < 0.001). The animals scored significantly higher proportions of decisions for the larger food piece when they were ball-shaped compared to when they were cube-shaped (Friedman ANOVA, Χ^2^ = 5.69, *p* < 0.05; Wilcoxon test, balls *versus* cubes: z = −2.31, *p* < 0.05) (Fig. 1).

Males and females did not differ significantly from each other in their performance (Mann-Whitney U-test, *p* > 0.05 with all size difference bins and with all three shapes). At the individual level, all animals scored at least 88% correct decisions, and the majority even 100% decisions, for the larger food piece when the size difference between the two food pieces was 71% or more (see Tables 1, 2 and 3 in the Supplemental material). This was true with cube-shaped food pieces as well as with ball-shaped and hemisphere-shaped food pieces. In contrast, hardly any animal scored more than 70% decisions for the larger food piece when the size difference between the two food pieces was less than 11%. This, too, was true with all three shapes of food pieces.

### Experiment 2: operant conditioning **test - visual size discrimination of wooden blocks**

All nine animals that participated in this experiment succeeded with reaching the learning criterion when presented with the first stimulus combination representing the largest size difference between the wooden blocks (30 *versus* 10 mm edge length). On average, they needed 6.3 ± 4.0 sessions for reaching the learning criterion, with the fastest-learning animals needing only 3 sessions and the slowest-learning animal needing 14 sessions. (Please note that the minimum number of sessions to reach the learning criterion was 3).

**Fig. 2:**
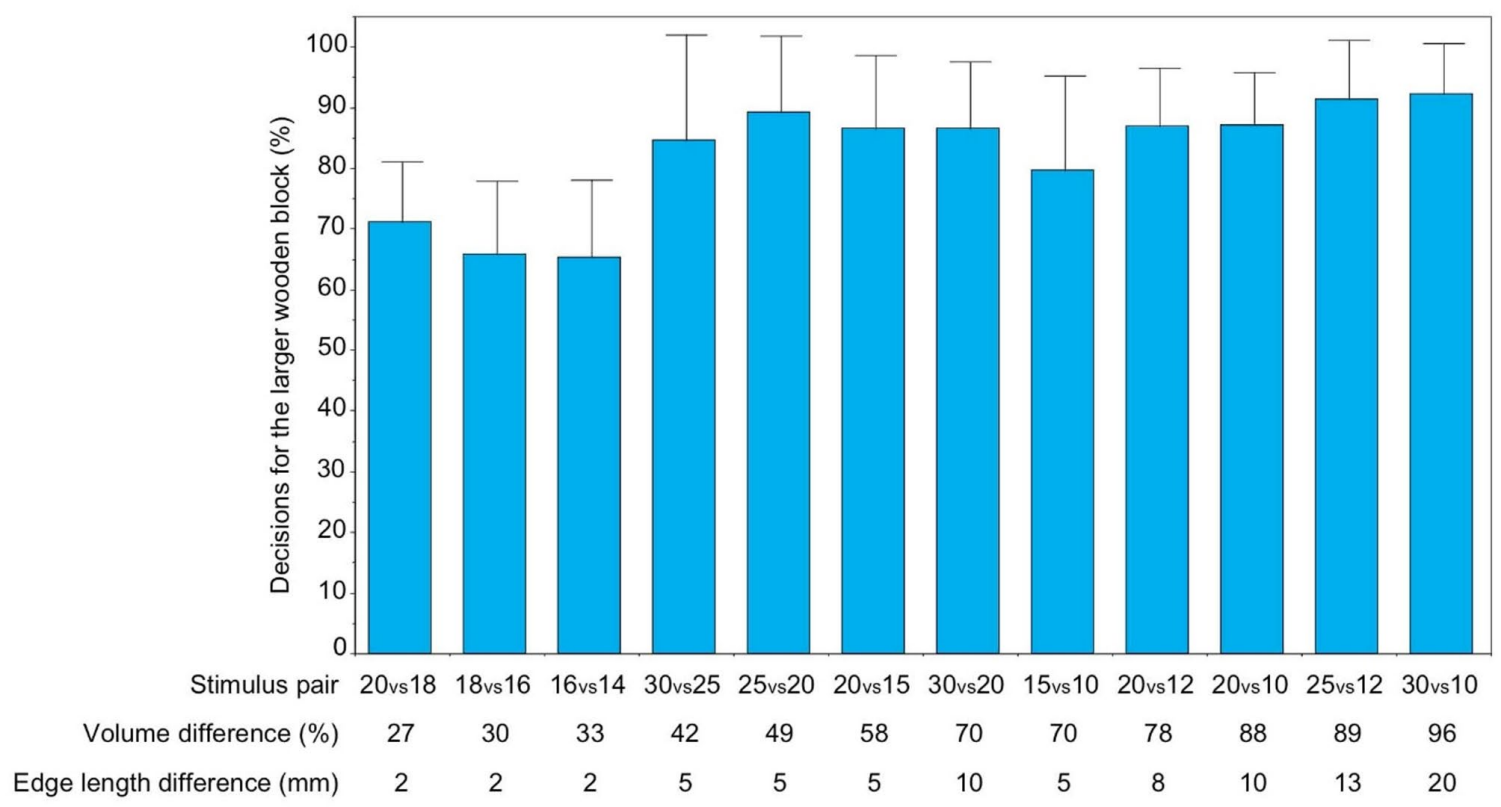
Mean (±SD) percentages of decisions for the larger one of two simultaneously presented wooden blocks for the group of spider monkeys (*n* = 9), subdivided by stimulus pairs.

As a group, the spider monkeys successfully discriminated between two simultaneously presented wooden blocks even with the smallest size difference tested (20 *versus* 18 mm edge length) corresponding to a volume difference of 27% (Binomial test, *p* < 0.01 with all 12 stimulus combinations tested) (Fig. 2). The proportion of correct decisions for the larger wooden block significantly correlated positively with size difference (Spearman test, r_s_ = 0.83 *p* < 0.01).

Males and females did not differ significantly from each other in their discrimination performance (Mann-Whitney U-test, *p* > 0.05 with all 12 stimulus combinations). At the individual level, all nine animals scored at least 80% correct decisions, and the majority even 100% correct decisions, with the largest size difference (30 *versus* 10 mm edge length) whereas only four animals scored 75% or more correct decisions with the smallest size difference (20 *versus* 18 mm edge length) (see Table 4 in the Supplemental material).

The absolute size of the wooden blocks did not systematically affect discrimination performance as the proportion of correct decisions did not differ significantly between the four stimulus combinations in which the wooden blocks differed by 5 mm in edge length (30 *versus* 25, 25 *versus* 20, 20 *versus* 15, and 15 *versus* 10 mm edge length) (Friedman ANOVA, Χ^2^ = 1.90, *p* > 0.05). The same was true with the three stimulus combinations in which the wooden blocks differed by 2 mm in edge length (20 *versus* 18, 18 *versus* 16, and 16 *versus* 14 mm edge length) (Friedman ANOVA, Χ^2^ = 2.67, *p* > 0.05) and with the two stimulus combinations in which the wooden blocks differed by 10 mm in edge length (30 *versus* 20, and 20 *versus* 10 mm edge length) (Wilcoxon test, z = −0.14, *p* > 0.05).

However, the four stimulus combinations in which the wooden blocks differed by 5 mm edge length (30 *versus* 25, 25 *versus* 20, 20 *versus* 15, and 15 *versus* 10 mm edge length) and the two stimulus combinations in which they differed by 10 mm edge length (30 *versus* 20, and 20 *versus* 10 mm edge length), respectively, yielded significantly higher discrimination scores compared to the three stimulus combinations in which the wooden blocks differed by only 2 mm edge length (20 *versus* 18, 18 *versus* 16, and 16 *versus* 14 mm edge length) (Kruskal-Wallis test, H = 19.81, *p* < 0.01; post-hoc Dunn’s test, *p* < 0.01 with 2 *versus* 5 mm, and 2 *versus* 10 mm edge length pairs, and *p* > 0.05 with 5 *versus* 10 mm edge length pairs).

## Discussion

The results of the present study demonstrate that spider monkeys have a well-developed ability to visually discriminate between different sizes of food and of non-edible objects. They displayed a spontaneous preference for the larger one of two simultaneously presented pieces of food of the same kind when the size difference was 11% or larger. They also learned to choose the larger one of two simultaneously presented wooden cubes when their edge length differed by only 2 mm which was the smallest size difference tested.

### Experiment 1: spontaneous preference test - visual size discrimination of food items

Our finding that the spider monkeys displayed a spontaneous preference for the larger one of two food pieces of the same kind may not seem surprising as the optimal foraging theory predicts that evolution should favor individuals which succeed with maximizing their intake of critical nutrients and metabolic energy^1^. However, whereas numerous studies reported that fruit size plays an important role for food selection in members of all major primate taxa (strepsirrhines^33^:; platyrrhines^16^:; catarrhines^14^:; hominids^13^:), only very few studies so far determined the minimum size difference for which an animal displays a clear preference when allowed to choose between two food items of the same kind. Using an experimental design very similar to the one employed in the present study, chimpanzees (*Pan troglodytes*) were found to reliably prefer the larger one of two simultaneously presented cube-shaped pieces of banana when the size difference was 11% or larger^34^. This value is identical to the one found here with spider monkeys.

Spider monkeys and chimpanzees are both considered as primarily frugivorous and as ripe-fruit specialists^20,35^, with proportions of fruit in their diet ranging between 69 and 91% in the former^19,36^ and between 55 and 75% in the latter^37,38^, depending on the study site and season of the year. Considering that fruits are more patchily distributed both spatially and temporally compared to leaves, for example^29^, it seems reasonable to assume that differences in fruit size should be more important for frugivorous primates than differences in leaf size may be for folivorous primates. Future studies should therefore assess possible links between dietary specialization and the minimal size difference of food items for which primates display a clear preference.

It is interesting to note that the normal variation in size among fully ripe fruits of a given plant species has been reported to be at least 10%^39,40^. Accordingly, the minimum size difference of 11% between food pieces of the same kind for which the spider monkeys showed a clear preference is in the range of the normal size variation of the ripe fruits they feed on in the wild. This, in turn, suggests that spider monkeys may display an evolutionary adaptation in their pickiness with regard to intraspecific fruit size variation. This notion is also in line with our finding that the proportion of decisions for the larger food piece increased significantly with increasing size difference (Fig. 1).

We also found that the spider monkeys scored significantly higher proportions of decisions for the larger one of two food pieces when they were ball-shaped compared to when they were cube-shaped. As fruits consumed by primates are commonly ball-shaped or ovoid and hardly ever cube-shaped^41^ this should not be surprising. Nevertheless, this finding is not trivial as cube-shaped objects, or other three-dimensional objects with clear edges, may provide an additional visual cue to assess size differences that ball-shaped or ovoid objects are lacking^42,43^.

### Experiment 2: operant conditioning **test - visual size discrimination of wooden blocks**

We found that the spider monkeys as a group succeeded with discriminating between two simultaneously presented wooden blocks even when their edge length differed by only 2 mm which was the smallest size difference tested (Fig. 2). This corresponds to a difference of 27% in volume between a wooden block of 20 mm edge length and a wooden block of 18 mm edge length.

Previous studies using an experimental design similar to the one employed here reported that human subjects (*Homo sapiens*), long-tailed macaques (*Macaca fascicularis*), chimpanzees (*Pan troglodytes*), bonobos (*Pan paniscus*), rhesus macaques (*Macaca mulatta*), and olive baboons (*Papio anubis*) succeeded with discriminating between non-edible cube-shaped objects which differed by 14, 14, 18, 19, 25, and 30%, respectively, in volume (Table 1).

**Table 1:** Minimum volume difference (%) and minimum edge length difference (mm) between three-dimensional objects of the same shape that various primate species visually discriminate above chance level. [x] present study.

Species	Minimum volume difference (%)	Minimum edge length difference (mm)	Object features	Ref.
Spider monkeys	11*		ball-shaped, edible	[x]
Spider monkeys	11*		cube-shaped, edible	[x]
Spider monkeys	11*		hemisphere-shaped, edible	[x]
Chimpanzee	11*		cube-shaped, edible	^34^
Spider monkeys	27	2	cube-shaped, non-edible	[x]
Human subjects	14	3	cube-shaped, non-edible	^44^
Long-tailed macaques	14	3	cube-shaped, non-edible	^44^
Chimpanzees	18	4	cube-shaped, non-edible	^44^
Bonobos	19	4	cube-shaped, non-edible	^44^
Olive baboons	30	6	cube-shaped, non-edible	^44^
Rhesus macaques	25	15	cube-shaped, non-edible	^45^

*Please note that these values are based on a spontaneous preference test whereas all other values are based on operant conditioning tests.

At first sight, this suggests that spider monkeys perform poorer in visual size discrimination of non-edible cube-shaped objects than the majority of other primate species tested so far. However, two caveats should be considered in this context:

Firstly, whereas the values mentioned above for the other primate species represent the smallest difference in volume that the animals were able to successfully discriminate, the value reported here for the spider monkeys only represents the smallest difference in volume that was tested. Accordingly, we cannot exclude the possibility that the spider monkeys may also have succeeded with discriminating between even smaller volume differences.

Secondly, all previous studies presented the animals with cubes that were much larger in size compared to the cubes employed in the present study. Schmitt et al.^44^, for example, used cubes with an edge length between 44 and 58 mm, and Brown et al.^45^ used cubes with an edge length between 76 and 133 mm, whereas our cubes ranged in edge length between 10 and 30 mm. Accordingly, the minimum difference in edge length that the primates tested by the above-mentioned authors successfully discriminated ranged between 3 mm (with human subjects and long-tailed macaques) and 6 mm (with olive baboons), and was 15 mm with the rhesus macaques (Table 1). Thus, we cannot conclude that the spider monkeys performed poorer in visually discriminating between non-edible cube-shaped objects than other primate species. Rather, they succeeded with cubes of a smaller edge length difference (2 mm) compared to the other primate species.

Our rationale for using smaller cube-shaped objects than the previous studies was that we felt it important to present the spider monkeys with objects of a size that is similar to the size of the fruits that they mainly feed on in the wild^19,20^ and thus represent a size range that is ecologically relevant in the context of food selection.

We also found that all nine spider monkeys that participated in the second experiment succeeded with learning the concept that choosing the larger one of two simultaneously presented non-edible cubes is rewarded. This is not trivial considering that eight out of eight gorillas, four out of eight long-tailed macaques, four out of nine olive baboons, and one out of five chimpanzees failed to learn this concept in the study by Schmitt et al.^44^ which used an operant conditioning procedure similar to the one employed here. The authors discuss that the failure of all gorillas in this visual size discrimination task may correlate with them being the least frugivorous of the species tested and that frugivory may have favored the evolution of the ability to perform fine size discriminations. Our finding that none of the highly frugivorous spider monkeys of the present study failed with learning the concept is in line with this notion.

Related to the learning success of the spider monkeys, we also found that they only needed 6.3 sessions (corresponding to 63 trials) on average to reach the learning criterion in this task. This is markedly faster than the 17.7 sessions (corresponding to 177 trials) that the same animals needed on average to reach the same learning criterion in a previous study where they had to learn to discriminate between two-dimensional objects^28^. Of course, we cannot exclude the possibility that this previous experience contributed to the faster learning of the animals in the present study. However, our finding also fits to the notion that animals generally learn faster to visually discriminate between three-dimensional objects than between two-dimensional objects, presumably because the former offer depth cues, and thus an additional object feature, which animals are likely to use for object discrimination, and which the latter are lacking^46^. This difference in discrimination learning speed with two- and three-dimensional objects, respectively, has been reported both for primates such as rhesus macaques^47^ and capuchins^48^ as well as for non-primate mammals^49^ and birds^50^.

Unfortunately, the learning speed in terms of number of trials or sessions needed to reach the learning criterion was not reported in the earlier studies on size discrimination of cube-shaped objects in the primates mentioned above^44,45^. Thus, we cannot draw any conclusion as to how spider monkeys rank among primates with regard to learning speed in visual three-dimensional object discrimination tasks.

### Comparison between experiments 1 and 2

It is commonly agreed that discrimination tests based on spontaneous preferences provide only a conservative approximation of an animal’s ability to distinguish between two options because small size differences between food items, for example, may not be behaviorally relevant enough to elicit a clear preference despite the animal’s sensory ability to perceive and recognize the size difference in question^30^. Operant conditioning procedures, in contrast, usually allow us to assess the limits of a sensory capability such as visual size discrimination^51^.

At first sight, it therefore seems counterintuitive that the spider monkeys of the present study reliably preferred the larger one of two simultaneously presented pieces of food when these differed by only 11% in volume (Fig. 1) whereas they already had some difficulty, although still performed above chance level, when being food-rewarded for choosing the larger one of two non-edible wooden cubes which differed by 27% in volume (Fig. 2). However, here, too, two caveats should be considered:

Firstly, as mentioned above, the value of 27% only represents the smallest difference in volume that was tested in the operant conditioning test using wooden cubes. Thus, it could well be that the spider monkeys may also have succeeded with discriminating between even smaller volume differences.

Secondly, it is obvious that the sight and smell of food has immediate behavioral relevance and thus can be a strong motivator for an animal in all kinds of test situations whereas the potential behavioral relevance of non-edible items such as wooden cubes first has to be learned via association building. Our spontaneous preference test allowed the spider monkeys to see and possibly also smell the food pieces presented to them even prior to their decision making while the wooden cubes presented in the operant conditioning test provided neither a visual nor an olfactory cue of the food reward that was provided after the animal had made its decision. Thus, it seems reasonable to assume that both the sensory availability of the food and the immediacy of consuming the food reward upon deciding for one of the options may have helped the animals to pay more attention to small size differences during the spontaneous preference test compared to the operant conditioning test.

Numerous studies support the notion that both the behavioral relevance of food in a test situation^52,53^ as well as the immediacy of the food reward upon decision making^51,54^ may play a crucial role for the outcome of discrimination tests. In line with these considerations, and in parallel to our findings with spider monkeys, chimpanzees have also been found to discriminate between smaller size differences with cube-shaped food pieces than with cube-shaped wooden blocks (Table 1).

In summary, we found that spider monkeys have a well-developed ability to visually discriminate between different sizes of food and of non-edible three-dimensional objects which is at least as good as that of other nonhuman primate species tested previously on similar tasks.

## Electronic supplementary material

Below is the link to the electronic supplementary material.

Supplementary Material 1

## Data Availability

All relevant data are presented in the manuscript. Detailed data are available from the corresponding author upon reasonable request.
