# Supplementary material for: The ability of captive spider monkeys, Ateles geoffroyi, to visually discriminate between different sizes of food and of non-edible objects
Source: Sci Rep. 2025 Jun 20;15:20211. doi: 10.1038/s41598-025-06479-8 (PMC12181230; doi:10.1038/s41598-025-06479-8)
Supplement: Supplementary file 1 — Supplementary Material 1 [file 41598_2025_6479_MOESM1_ESM.docx]

**Supplementary Table 1.** Percentages of decisions for the larger one of two simultaneously presented cube-shaped food pieces made by each individual animal, subdivided by increasing size difference. The ratios in brackets are the numbers of decisions for the larger and the smaller cube-shaped food piece, respectively. The percentages of decisions that significantly differed from chance are indicated by asterisks (Binomial test, *p < 0.05).

| **% Size difference** | **0-10** | **11-20** | **21-30** | **31-40** | **41-50** | **51-60** | **61-70** | **71-80** | **81-90** |
| --- | --- | --- | --- | --- | --- | --- | --- | --- | --- |
| Chanel | 67 (4:2) | 75 (6:2) | 87* (13:2) | 94* (16:1) | 83* (15:3) | 79* (15:4) | 95* (18:1) | 100* (11:0) | 100* (7:0) |
| Bart | 63 (5:3) | 75 (6:2) | 91* (10:1) | 96* (23:1) | 95* (19:1) | 81* (13:3) | 90* (9:1) | 100* (14:0) | 100* (9:0) |
| Homero | 40 (2:3) | 87* (13:2) | 80* (12:3) | 100* (11:0) | 100* (18:0) | 100* (20:0) | 100* (8:0) | 100* (18:0) | 100* (10:0) |
| Frida | 50 (1:1) | 63 (5:3) | 50 (4:4) | 77* (10:3) | 60 (12:8) | 59 (13:9) | 100* (17:0) | 71* (12:5) | 92* (12:1) |
| Trilce | 75 (3:1) | 83* (10:2) | 90* (9:1) | 90* (18:2) | 85* (17:3) | 100* (13:0) | 100* (18:0) | 100* (12:0) | 100* (11:0) |
| Thalia | 50 (2:2) | 67 (8:4) | 100* (14:0) | 94* (17:1) | 94* (16:1) | 100* (19:0) | 100* (8:0) | 100* (15:0) | 100* (13:0) |
| Juanis | 67 (2:1) | 55 (6:5) | 80* (8:2) | 71* (10:4) | 50 (10:10) | 79* (19:5) | 75* (12:4) | 94* (17:1) | 100 (4:0) |
| Primo | 67 (4:2) | 77* (10:3) | 63 (10:6) | 81* (13:3) | 57 (8:6) | 100* (15:0) | 94* (15:1) | 95* (18:1) | 100 (5:0) |
| Nena | 60 (3:2) | 100* (7:0) | 78* (7:2) | 80* (12:3) | 72* (13:5) | 67* (14:7) | 76* (13:4) | 83* (15:3) | 90* (9:1) |
| Sorujo | 40 (2:3) | 59 (10:7) | 78* (7:2) | 75* (15:5) | 81* (13:3) | 81* (13:3) | 93* (14:!) | 93* (14:1) | 100* (7:0) |
| mean±SD | 57.9±12.2 | 74.1±13.7* | 79.7±14.4* | 85.8±10.2* | 77.7±17.3* | 84.6±14.9* | 92.3±9.5* | 93.6±9.6* | 98.2±3.8* |

**Supplementary Table 2.** Percentages of decisions for the larger one of two simultaneously presented ball-shaped food pieces made by each individual animal, subdivided by increasing size difference bins. The ratios in brackets are the numbers of decisions for the larger and the smaller ball-shaped food piece, respectively. The percentages of decisions that significantly differed from chance are indicated by asterisks (Binomial test, *p < 0.05).

| **% Size difference** | **0-10** | **11-20** | **21-30** | **31-40** | **41-50** | **51-60** | **61-70** | **71-80** |
| --- | --- | --- | --- | --- | --- | --- | --- | --- |
| Chanel | 63 (12:7) | 75* (18:6) | 81* (13:3) | 67 (4:2) | 100*(23:0) | 94* (17:1) | 100* (13:0) | 100 (1:0) |
| Bart | 61 (11:7) | 91* (21:2) | 85* (11:2) | 100* (11:0) | 100* (21:0) | 100* (18:0) | 100* (14:0) | 100 (2:0) |
| Homero | 60 (24:16) | 78* (14:4) | 100 (4:0) | 100* (7:0) | 100* (18:0) | 100* (20:0) | 100* (11:0) | 100 (2:0) |
| Frida | 39 (7:11) | 50 (12:12) | 82* (9:2) | 71 (10:4) | 82* (14:3) | 100* (18:0) | 100* (17:0) | 100 (1:0) |
| Trilce | 74* (17:6) | 82* (14:3) | 91* (10:1) | 91* (10:1) | 94* (17:1) | 95* (18:1) | 100* (18:0) | 100 (1:0) |
| Thalia | 67 (30:15) | 73 (11:4) | 100 (1:0) | 86* (6:1) | 84* (21:4) | 96* (23:1) | 100 (1:0) | 100 (2:0) |
| Juanis | 70 (16:7) | 85* (11:2) | 77* (10:3) | 78* (14:4) | 80* (16:4) | 100* (18:0) | 92* (12:1) | 100 (2:0) |
| Primo | 66 (27:14) | 81* (13:3) | 83* (5:1) | 88* (7:1) | 92* (24:2) | 95* (19:1) | 100 (2:0) | 100 (1:0) |
| Nena | 54 (26:22) | 70 (7:3) | 50 (2:2) | 100* (5:0) | 82* (18:4) | 96* (24:1) | 100* (5:0) | 100 (1:0) |
| Sorujo |  |  | 67 (2:1) | 67 (2:1) | 94 (15:1) | 100* (19:0) | 100* (5:0) | 100 (1:0) |
| mean±SD | 61.6±10.3 | 76.1±11.7* | 81.6±14.9* | 84.8±13.4* | 90.8±8.1* | 97.6±2.6* | 99.2±2.5* | 100.0±0.0* |

**Supplementary Table 3.** Percentages of decisions for the larger one of two simultaneously presented hemisphere-shaped food pieces made by each individual animal, subdivided by increasing size difference bins. The ratios in brackets are the numbers of decisions for the larger and the smaller hemisphere-shaped food piece, respectively. The percentages of decisions that significantly differed from chance are are indicated by asterisks (Binomial test, *p < 0.05).

| **% Size difference** | **0-10** | **11-20** | **21-30** | **31-40** | **41-50** | **51-60** | **61-70** | **71-80** |
| --- | --- | --- | --- | --- | --- | --- | --- | --- |
| Chanel | 38 (3:5) | 78 (7:2) | 85* (11:2) | 100* (8:0) | 83* (5:1) | 100* (6:0) | 100* (7:0) | 100 (3:0) |
| Bart | 71 (5:2) | 82* (9:2) | 100* (10:0) | 100* (11:0) | 100* (10:0) | 100* (7:0) | 100 (2:0) | 100 (2:0) |
| Homero | 36 (4:7) | 71 (12:5) | 89* (8:1) | 80 (4:1) | 86* (6:1) | 83* (5:1) | 100 (3:0) | 100 (2:0) |
| Frida | 60 (3:2) | 56 (9:7) | 50 (3:3) | 50 (3:3) | 91* (10:1) | 100* (11:0) | 100 (2:0) | 100 (3:0) |
| Trilce | 67 (4:2) | 69 (11:5) | 79* (11:3) | 80 (4:1) | 86* (6:1) | 100* (5:0) | 100 (4:0) | 100 (3:0) |
| Thalia | 60 (3:2) | 75 (9:3) | 90* (9:1) | 80 (4:1) | 83* (10:2) | 91* (10:1) | 100 (2:0) | 100 (3:0) |
| Juanis |  | 77 (10:3) | 100 (4:0) | 100* (5:0) | 100* (5:0) | 100* (14:0) | 100* (8:0) | 100 (2:0) |
| Primo | 44 (4:5) | 75* (12:4) | 80 (4:1) | 86* (6:1) | 83* (5:1) | 91* (10:1) | 100 (3:0) | 100 (3:0) |
| Nena | 50 (4:4) | 62 (8:5) | 80 (4:1) | 83* (5:1) | 86* (6:1) | 78* (7:2) | 75 (3:1) | 88* (7:1) |
| mean±SD | 53.3±13.2 | 71.7±8.2* | 83.7±14.9* | 84.3±15.7* | 88.7±6.9* | 93.7±8.5* | 97.2±8.3* | 98.7±4.0* |

**Supplementary Table 4.** Percentages of decisions for the larger one of two simultaneously presented wooden blocks made by each individual animal, subdivided by increasing volume differences. The ratios in brackets are the numbers of decisions for the larger and the smaller wooden block, respectively. The percentages of decisions that significantly differed from chance are indicated by asterisks (Binomial test, *p < 0.05).

| **Stimulus pair** | **20vs18** | **18vs16** | **16vs14** | **30vs25** | **25vs20** | **20vs15** | **30vs20** | **15vs10** | **20vs12** | **20vs10** | **25vs12** | **30vs10** |
| --- | --- | --- | --- | --- | --- | --- | --- | --- | --- | --- | --- | --- |
| **Volume difference (%)** | **27** | **30** | **33** | **42** | **49** | **58** | **70** | **70** | **78** | **88** | **89** | **96** |
| **Edge length difference (mm)** | **2** | **2** | **2** | **5** | **5** | **5** | **10** | **5** | **8** | **10** | **13** | **20** |
| Chanel | 75* (9:3) | 56 (5:4) | 67 (6:3) | 100* (9:0) | 100* (11:0) | 100* (9:0) | 89* (8:1) | 75* (9:3) | 89* (8:1) | 100* (11:0) | 90* (9:1) | 100* (10:0) |
| Bart | 67 (8:4) | 67 (6:3) | 44 (4:5) | 100* (10:0) | 67 (6:3) | 89* (8:1) | 100* (9:0) | 91* (10:1) | 100* (9:0) | 100* (11:0) | 100* (9:0) | 100* (10:0) |
| Homero | 75* (9:3) | 56 (5:4) | 44 (4:5) | 56 (5:4) | 91* (10:1) | 89* (8:1) | 73 (8:3) | 92* (11:1) | 75 (6:2) | 89* (8:1) | 73 (8:3) | 80* (8:2) |
| Frida | 67 (8:4) | 56 (5:4) | 78* (7:2) | 83* (8:1) | 100* (10:0) | 100* (9:0) | 100* (11:0) | 83* (10:2) | 91* (10:1) | 78* (7:2) | 89* (8:1) | 90* (9:1) |
| Trilce | 92* (11:1) | 78* (7:2) | 56 (5:4) | 82* (9:2) | 100* (10:0) | 89* (8:1) | 91* (10:1) | 100* (10:0) | 91* (10:1) | 78* (7:2) | 100* (9:0) | 100* (10:0) |
| Thalia | 67 (8:4) | 89* (8:1) | 33 (3:6) | 100* (9:0) | 73 (8:3) | 89* (8:1) | 73 (8:3) | 58 (7:5) | 78* (7:2) | 89* (8:1) | 100* (10:0) | 80* (8:2) |
| Primo | 67 (6:3) | 78* (7:2) | 75* (9:3) | 89* (8:1) | 90* (9:1) | 89* (8:1) | 91*(10:1) | 92* (11:1) | 80* (8:2) | 89* (8:1) | 100* (10:0) | 90* (9:1) |
| Nena | 56 (5:4) | 56 (5:4) | 73 (8:3) | 56 (5:4) | 82* (9:2) | 67 (6:3) | 73 (8:3) | 58 (7:5) | 100* (9:0) | 78* (7:2) | 80* (8:2) | 90* (9:1) |
| Sorujo | 75* (9:3) | 56 (5:4) | 44 (4:5) | 89* (8:1) | 100* (11:0) | 67 (6:3) | 89* (8:1) | 67 (8:4) | 78* (7:2) | 82* (9:2) | 90* (9:1) | 100* (10:0) |
| mean±SD | 71.2±9.9* | 65.8±12.1* | 65.2±12.9* | 84.6±17.4* | 89.2±12.6* | 86.6±12.0* | 86.6±11.0* | 79.6±15.7* | 86.9±9.5* | 87.0±8.8* | 91.3±9.8* | 92.2±8.3* |
